# Transition to Virtual Reflection: Narrative Medicine during COVID-19

**DOI:** 10.15694/mep.2020.000112.1

**Published:** 2020-05-26

**Authors:** Yoshiko Iwai, Penelope Lusk

**Affiliations:** 1 Columbia University

**Keywords:** COVID-19, medical education, Narrative Medicine, online learning, virtual education, Zoom, pre-medical education, health humanities

## Abstract

This article was migrated. The article was marked as recommended.

Narrative medicine workshops are typically conducted in person and provide medical professionals and students with reflective spaces. During the COVID-19 pandemic, in-person workshops at one university were cancelled and moved online following social distancing measures. Narrative medicine workshop facilitators were challenged to transfer workshops online, while still encouraging creative reflection as the pandemic impacted participants’ professional and personal lives. One workshop for pre-medical students at the university moved online to Zoom, the standard platform for all university courses. The workshop length was shortened and the curriculum re-focused on creative texts and personal wellbeing. Participants responded positively to Zoom workshops although fewer individuals participated overall. Most participants were able to successfully use the platform although there were difficulties regarding WiFi and connection. Despite challenges, these workshops function in virtual spaces and provide an important opportunity for programs to integrate virtual sessions for wellness and reflection during a time of pandemic.

## Introduction

Traditional narrative medicine workshops, typically conducted in person, provide medical professionals and students with reflective spaces ( Charon, 2001) ( Milota *et al*., 2019). Due to social distancing in the wake of COVID-19, workshops led by facilitators from Columbia University’s Narrative Medicine master’s program were cancelled or moved online. Narrative medicine workshops, led by trained facilitators, follow a standard structure: participants read a text to spark discussion around personal wellbeing or ethical questions in medicine, then participate in a writing exercise and share their responses ( Winkel, 2016). With COVID-19, facilitators were challenged to transfer workshops online, while encouraging creative reflection as the pandemic impacted participants’ professional and personal lives.

As a case study, we explore a series of six 90 minute workshops for pre-medical students at one elite university who applied for and committed to attend the series. Social distancing measures took effect three weeks into the series.

## Virtual Workshops: Changes and Challenges

Following university closure, narrative medicine workshops moved online to Zoom, the standard platform for all university courses. The workshop was led by two facilitators (YI and PL) who used institutional Zoom accounts. With the transition online, there were three main changes that were implemented to respond to COVID-19: 1) the series attendance requirement was waived, 2) the last two workshops were changed from 90 to 60 minutes, and 3) the curriculum was re-organized to focus discussions on creative texts-poem, mixed-media essay, painting-and personal wellbeing, to lessen cognitive load and foster a supportive space.

Our primary concerns around virtual narrative medicine workshops were participant interest, attention, participation, and maintaining a safe space. Online workshops could potentially impact participants’ affective experience, as “it is in the contexts of embodied relation that significant differences between online and offline educational experience begin to appear” ( Friesen, 2011). We saw that compared to in-person workshops, attendance fell by half while participant engagement varied. While participants had minimal difficulty using Zoom technology because it was standard across the university, holding workshops on Zoom also made it difficult for students to focus. We saw decreased participation during the first online session. To address this, facilitators shortened the workshop to 60 minutes, and saw both student attention and participation increase. Additionally, we encouraged students to turn on their video and physically raise their hands, as opposed to the virtual “hand raising” function, which increased student engagement.

We also found that emphasizing creative texts contributed to the safe “classroom” environment. In light of COVID-19, we hoped our workshops would provide either, or *both*, a place of respite from the pandemic, or a place to actively reflect on the pandemic. We discussed creative materials including Mary Oliver’s poem “Wild Geese” and Edward Hopper’s painting “Morning Sun,” followed by personal writing responses. Students who shared their written responses received feedback from participants and facilitators, which increased overall engagement and helped build community, despite the mediation of a screen. Facilitators initially planned on using the breakout room function to encourage small group discussion on the creative materials, however, the decreased workshop size led facilitators to conduct the discussion following a creative material altogether. Had the class size exceeded eight, breakout room functions would have been used based on successful dyad discussions during in-person sessions.

## Conclusion

While medical education involves many hours spent online, curriculums often lack spaces for personal reflection, writing, or discussion ( Branch *et al.*, 2001) ( Karnieli-Miller *et al.*, 2010) ( Pedersen, 2010). With our virtual narrative medicine workshops, participants reported that they appreciated workshop continuity and enjoyed participating despite the online setting. Participants and facilitators occasionally experienced WiFi issues, although most were able to troubleshoot and return to the session. However, the drop in attendance may be partly attributable to students’ limited access to WiFi, electronic devices, and bandwidth.

While we experienced challenges with the initial movement online, we predict such workshops will continue to be positively received and provide constructive, accessible spaces for pre-medical and medical students and professionals during, and after, the pandemic. As Nellie Hermann, a narrative medicine scholar, writes: “Over the years in narrative medicine we have come to see more and more that our work is about reawakening the creativity that lives in all of us. When we go into a room and lead others in an exercise of reading and writing, we are encouraging everyone in that room to be creative: to put down their rigidly held convictions and engage in an exercise where there is no “right” answer, to allow themselves to be swept into something different, something they may not be able to see the end result of” ( Charon *et al*., 2016). Ultimately, we hope that virtual narrative medicine workshops during COVID-19 encourage participants and facilitators to reflect and process our present reality, while fostering spaces to nurture creativity to current and future caregivers.

## Take Home Messages

• Continuation of narrative medicine workshops online is important in medical education.
• Waiving attendance and decreasing workshop duration are effective methods for online workshops.
• Focusing on creative materials encourages reflection on the present COVID-19 while maintaining a safe space.

## Notes On Contributors

Yoshiko Iwai, MS, MFA, is a graduate of Columbia University’s programs in Narrative Medicine and Creative Nonfiction. She is published in Scientific American, Academic Medicine, Journal of Medical Humanities, among others.

ORCiD: https://orcid.org/0000-0001-9759-384X

Penelope Lusk, MS, is a graduate of Columbia University’s program in Narrative Medicine and the project associate for the Program for Medical Education Innovations and Research, NYU School of Medicine. She is published in Journal of General Internal Medicine, Diagnosis, and Medical Education.

ORCiD: https://orcid.org/0000-0001-6202-9376

## References

[ref1] Branch W. T. Jr. Kern D. Haidet P. Weissmann P. (2001) Teaching the human dimensions of care in clinical settings. JAMA. 286(9),1067–1074. 10.1001/jama.286.9.1067 11559292

[ref2] Charon R. (2001) Narrative Medicine: Form, Function, and Ethics. Annals of Internal Medicine. 134(1),83–87. 10.7326/0003-4819-134-1-200101020-00024 11187429

[ref3] Charon R. DasGupta S. Hermann N. Irvine C. (2016) The Principles and Practice of Narrative Medicine. New York, NY: Oxford University Press. 10.1093/med/9780199360192.001.0001

[ref4] Friesen N. (2011) The Place of the Classroom and the Space of the Screen: Relational Pedagogy and Internet Technology. New Literacies and Digital Epistemologies. New York, NY: Peter Lang Publishing Inc.

[ref5] Karnieli-Miller O. Vu T. R. Holtman M. C. Clyman S. G. (2010) Medical students’ professionalism narratives: A window on the informal and hidden curriculum. Academic Medicine. 85(1),124–133. 10.1097/ACM.0b013e3181c42896 20042838

[ref6] Milota M. M. van Thiel W. J. G. M.and van Delden M. J. J.(2019) Narrative medicine as a medical education tool: A systematic review. Medical Teacher. 41(7),802–810. 10.1080/0142159X.2019.1584274 30983460

[ref7] Pedersen R. (2010) Empathy development in medical education - A critical review. Medical Teacher. 32(7),593–600. 10.3109/01421590903544702 20653383

[ref8] Winkel A. F. (2016) Narrative Medicine: A Writing Workshop Curriculum for Residents. MedEdPORTAL. 12. 10.15766/mep_2374-8265.10493 PMC6440423 30984835

