# Supplementary material for: Transition to Virtual Reflection: Narrative Medicine during COVID-19
Source: MedEdPublish (2016). 2020 May 26;9:112. [Version 1] doi: 10.15694/mep.2020.000112.1 (PMC10702644; doi:10.15694/mep.2020.000112.1)
Supplement: Peer review history

# Reviewer report: Reviewer response for version 1

**DOI:** 10.21956/mep.20098.r30974

**Published:** 2020-07-05

**Authors:** Irina Markovina

**Affiliations:** 1 Sechenov First Moscow State Medical University (Sechenov University)

This review has been migrated. The reviewer awarded 4 stars out of 5 I find the theme of the article important in several respects. Both the pandemic and online training are currently hot topics in higher education, and medical education is not an exception. The article is well structured and discusses interesting and relevant experience. I believe that experience shared by the authors may help other educators to be more efficient in giving virtual workshops in terms of both organization and content development. One of the most positive conclusions I arrived at was that it is important for the educator to reflect on the current experience, make conclusions and introduce changes in the ongoing education process. Another idea I would like to emphasize and support is that difficult times of COVID-19 pandemic should be accompanied with creativity when training health care professionals. This current difficult situation with the pandemic has already demonstrated that Humanities help. However, I wish more examples of the materials discussed at virtual workshops were given. There seems to be some kind of an imbalance between the organizational issues covered and the creative content of the workshops. I would agree with the authors that we should look for the best way to combine in-person sessions with virtual classes for the benefit of our students.

Reviewer Expertise:

NA

No decision status is available

# Reviewer report: Reviewer response for version 1

**DOI:** 10.21956/mep.20098.r30973

**Published:** 2020-06-01

**Authors:** P Ravi Shankar

**Affiliations:** 1 American International Medical University

This review has been migrated. The reviewer awarded 4 stars out of 5 This is an interesting write up about online narrative medicine workshops. The authors have mentioned the challenges of the workshop and of moving sessions online. In subjects like the medical humanities where physical face-to-face interaction is very important and the physical and emotional setting of the room plays an important role moving online may not achieve the same level of belonging. Interest and interaction may be difficult to achieve in an online space but the COVID-19 pandemic calls on us to try. How many students participated in the online workshops? Did students write on their computers or did they use paper and pencil? I am surprised that you have internet issues in New York, United States. In many other parts of the world it is an important issue to be considered while planning online workshops. I would be interested in hearing more about these online workshops as they progress. Do you plan to continue them post-COVID as the situation normalizes?

Reviewer Expertise:

NA

No decision status is available

# Reviewer report: Reviewer response for version 1

**DOI:** 10.21956/mep.20098.r30972

**Published:** 2020-05-26

**Authors:** Elisabeth Schlegel

**Affiliations:** 1 Donald and Barbara Zucker School of Medicine at Hofstra/Northwell

This review has been migrated. The reviewer awarded 3 stars out of 5 I am thanking the authors for the effort of transferring narrative medicine online. Challenges for students might include overcoming shyness to speak on camera (but might quickly adapt). I found that your efforts are aligned with Moore’s Theory of Transactional Distance (1997). According to Moore, the nature of the transaction between educators and learners in distance learning need to take into account three factors, (1) Dialogue; (2) Structure; and (3) Learner Autonomy. Reading your article I found the effort easily establishes an online community of peer-teachers and learners, sharing reflective thoughts. This is especially fruitful for pre-med students who need to get into the habit of cultivating reflection, with topics of ethics and wellbeing supporting tenets of professionalism. I encourage to offer repeat workshops of personal reflection and to collect data through a mixed-methods approach.Ref: Moore, M. (1997). Theory of transactional distance. In D. Keegan (Ed.), Theoretical principles of distance education (pp. 22–38). New York: Routledge.

Reviewer Expertise:

NA

No decision status is available
